# Polyomic tools for an emerging livestock parasite, the rumen fluke *Calicophoron daubneyi*; identifying shifts in rumen functionality

**DOI:** 10.1186/s13071-018-3225-6

**Published:** 2018-12-04

**Authors:** Kathryn M. Huson, Russell M. Morphew, Nathan R. Allen, Matthew J. Hegarty, Hillary J. Worgan, Susan E. Girdwood, Eleanor L. Jones, Helen C. Phillips, Martin Vickers, Martin Swain, Daniel Smith, Alison H. Kingston-Smith, Peter M. Brophy

**Affiliations:** Institute of Biological, Environmental & Rural Sciences (IBERS), Aberystwyth University, Penglais, Ceredigion, Aberystwyth, SY23 3DA UK

**Keywords:** *Calicophoron daubneyi*, Transcriptome, Paramphistome, Proteomics, *In vitro*, Rumen fermentation

## Abstract

**Background:**

Diseases caused by parasitic flatworms of rumen tissues (paramphistomosis) are a significant threat to global food security as a cause of morbidity and mortality in ruminant livestock in subtropical and tropical climates. *Calicophoron daubneyi* is currently the only paramphistome species commonly infecting ruminant livestock in temperate European climates. However, recorded incidences of *C. daubneyi* infection in European livestock have been increasing over the last decade. Whilst clinical paramphistomosis caused by adult worms has not been confirmed in Europe, fatalities have been attributed to severe haemorrhagic enteritis of the small intestine resulting from the migration of immature paramphistomes. Large numbers of mature adults can reside in the rumen, yet to date, the impact on rumen fermentation, and consequently on productivity and economic management of infected livestock, have not been resolved. Limited publicly available nucleotide and protein sequences for *C. daubneyi* underpin this lack of biological and economic understanding. Here we present for the first time a *de novo* assembled transcriptome, with functional annotations, for adult *C. daubneyi*, which provides a reference database for protein and nucleotide sequence identification to facilitate fundamental biology, anthelmintic, vaccine and diagnostics discoveries.

**Results:**

This dataset identifies a number of genes potentially unique to *C. daubneyi* and, by comparison to an existing transcriptome for the related *Paramphistomum cervi*, identifies novel genes which may be unique to the paramphistome group of platyhelminthes. Additionally, we present the first coverage of the excretory/secretory and soluble somatic proteome profiles for adult *C. daubneyi* and identify the release of extracellular vesicles from adult *C. daubneyi* parasites during *in vitro*, *ex-host* culture. Finally, we have performed the first analysis of rumen fluke impacting upon rumen fermentation parameters using an *in vitro* gas production study resulting in a significant increase in propionate production.

**Conclusions:**

The resulting data provide a discovery platform (transcriptome, proteomes, EV isolation pipeline and *in vitro* fermentation system) to further study *C. daubneyi*-host interaction. In addition, the acetate: propionate ratio has been demonstrated to decrease with rumen fluke infection suggesting that acidotic conditions in the rumen may occur.

**Electronic supplementary material:**

The online version of this article (10.1186/s13071-018-3225-6) contains supplementary material, which is available to authorized users.

## Background

Parasitic helminth infections of livestock are a global threat to food security, sustainable agriculture and animal welfare. Significant financial losses are incurred by individual livestock producers and national/international agricultural economies annually due to the costs associated with animal morbidity, mortality, production loss and anthelmintic treatment [1, 2]. Rumen fluke infection, or paramphistomosis, is a significant cause of morbidity and mortality in ruminant livestock in subtropical and tropical climates [3, 4] and is caused by several genera within the family Paramphistomidae. Paramphistomes have a complex indirect life-cycle, with a single intermediate snail host [5]. After ingestion by a ruminant host, metacercariae excyst in the duodenum and the immature parasites first move into the superficial mucosa of the intestine prior to migration up the alimentary tract to the rumen and reticulum. Mature paramphistomes appear generally well tolerated by host animals, with clinical disease frequently linked to immature rumen fluke causing significant damage to the mucosa of the duodenum [6, 7]. Upon *post-mortem* examination haemorrhagic inflammation of the duodenum may also be observed [8].

To date in Europe, paramphistomosis has not been considered clinically significant [9] and historically infection was believed to be less common [10, 11]. However, incidences of rumen fluke infection have dramatically increased in temperate areas of western Europe over the last few decades and many prevalence studies have identified rumen fluke as a common parasitosis of ruminant livestock in temperate European climates [12]. For example, of 100 farms in Wales 61% were identified as positive for *C. daubneyi* infection in sheep or cattle [13]. The apparent substantial prevalence of an infection previously regarded as less common has led to an urgent interest in this comparatively poorly studied helminth parasite. Thus, increased understanding of the impact from rumen fluke infection on animal production is crucial. To this end, *in vitro* rumen fermentation is established as a key technology to provide biological understanding of rumen dynamics [14, 15]. Ruminant animals rely on the microbial fermentation of feed and forage producing volatile fatty acids (VFAs) as their primary energy source [16]. Factors which impact on the profile of VFAs present in the rumen are known to impact on host nutrition with the ratios of the three major VFAs, produced *via* microbial fermentation in the rumen (acetate, propionate and butyrate) under a delicate balance.

Polyomics based technologies have allowed for significant expansion of our understanding of many aspects of parasitic helminth-host interaction biology in recent years [17–20]. To date, limited public available nucleotide and protein sequences for *C. daubneyi* hinder these functional genomic studies in this rapidly spreading livestock parasite. Thus, in the present study the molecular profile of the paramphistome *C. daubneyi* was revealed for the first time at both the transcript and the protein level *via* a functionally annotated *de novo* transcriptome and proteomic datasets for the excretory/secretory (ES) products and the soluble somatic proteome. The datasets presented here reveal evidence of predicted novel protein sequences from the transcriptome and in the proteomes and for the first time reveal the potential presence of extracellular vesicles released from adult *C. daubneyi* during *in vitro* culture. A gas production trial provides evidence of rumen fluke metabolism to produce increased propionate as supported by *C. daubneyi* transcriptome data that identifies genes involved in the propionate production pathway.

## Methods

### Species identification

In total during the study, paramphistomes were collected from 65 infected cattle from 22 farms around Wales, and 1 farm in Shropshire, England. Infected animals ranged in age from 19 months to 15 years and were of varying breeds from both beef and dairy production systems, further highlighting the widespread presence of this parasite.

For each infected bovine from which parasites were collected, DNA was extracted from 3 specimens using a Qiagen DNeasy® Blood and Tissue kit (Qiagen, Hilden, Germany) according to the manufacturer’s directions. DNA elutions were then subject to PCR amplification using *C. daubneyi* specific primers developed previously [21] targeting an 885 bp region of the cytochrome *c* oxidase subunit 1 (*cox*1) mitochondrial gene; Cd Cox1F (forward: 5'-TGG AGA GTT TGG CGT CTT TT-3') and Cd Cox1R (reverse: 5'-CCA TCT TCC ACC TCA TCT GG-3'). PCR products were visualised using gel electrophoresis on a 1% TAE agarose gel viewed under UV. Positive amplification with appropriate product size was given to confirm species identification. For individual rumen fluke used for RNAseq analysis, total RNA was isolated using an RNeasy (Qiagen) blood and tissue procedure according to the manufacturer’s instructions with 1 μg of total RNA used to create cDNA libraries for PCR amplification to confirm the species ID, using *cox*1 as stated above, prior to sequencing.

### Transcriptomics: sample collection, RNA isolation and sequencing

A natural rumen fluke parasite infection in a cow was identified immediately upon the opening of the rumen wall from a local abattoir (mid-Wales, UK). Individual parasite specimens from this single bovine host were rinsed briefly in sterile warm (39 °C) phosphate buffered saline (PBS) to remove large contaminating debris and immediately snap frozen in dry ice for transport. Samples were stored at -80 °C until RNA isolation. Initially, frozen samples were homogenised using a Qiagen TissueLyser LT (Qiagen). Total RNA was then extracted from 3 individual parasites using a RNeasy mini kit (Qiagen) according to the manufacturer’s directions, with an on-column DNase digestion step as directed in the RNeasy protocol. RNA quantity and integrity were measured using an Experion™ Automated Electrophoresis Station and RNA HighSens analysis kit (Bio-Rad, UK) with a RIN number of > 8 achieved for each sample. Total RNA (1 μg) was then used to purify polyadenylated (poly A+) mRNA according to the TruSeq RNA Sample Preparation v2 LS Workflow (Illumina, Cambridge, UK) using TruSeq RNA Sample Prep Kit v2 (Illumina) to prepare a 100 bp library for paired end sequencing. Each sample library was prepared with indexed adaptors as instructed in the Illumina workflow guide. Sequencing was performed on an Illumina HiSeq2500 platform according to standard protocols (Illumina).

### *De novo* assembly and bioinformatics

The raw Illumina data was demultiplexed and converted to sample fastq files using Illumina bcl2fastq software (version 1.8.3). Read quality was assessed using FastQC (http://www.bioinformatics.babraham.ac.uk/projects/fastqc/). Three quality control steps were carried out on the reads using Trimmomatic (v0.32 2) [22]; Truseq adaptor sequences were removed via a 13 base crop of the 5' end of the reads was carried out to resolve base bias identified by FastQC and the 3' end of the reads was cropped when the mean quality in a 4-base sliding window fell below a phred Q score of 20. Reads were assembled using Trinity (version date 2013-02-25) [23]. The resulting assembly file was functionally annotated using the Trinotate pipeline (V2.0) (https://trinotate.github.io/) and expression values for each contig, expressed as FPKM, were calculated using RSEM v1.2.25 [24]. Following RSEM analysis any unmapped reads (with an FPKM value of 0) were removed from further analyses. To visualise the gene ontology (GO) data for each contig the transcript ID and GO column data was extracted from the Trinotate output file and then loaded into Blast2GO® (V3.2) for visualisation. The top 50 expressed gene components identified by FPKM value were obtained by extracting the BLASTx UniProt identifier annotated to the longest isoform of each gene component ID and then uploading this to the UniProt mapping application (uniprot.org/mapping, accessed 03/12/2015) to obtain descriptive data on the protein matches, protein family, organism ID and associated GOslim information for each. For the top 50 expressed gene components identified by FPKM values which lacked any annotation, the longest isoform sequences for each was extracted from the assembly file and subjected to a BLASTn search of the NCBInr database in an attempt to match *C. daubneyi* sequences to any existing sequences with significant similarity. Further BLAST searches were performed against the SRA files available at SRA091604 (sheep), SRA039814 (goat) and SRA091607 (buffalo) with the transcriptome data for *P. cervi* [25] generated on an Ion Torrent™ PGM platform to identify sequences potentially unique to *C. daubneyi* or likely shared with other paramphistomes. To obtain a picture of the most active high-level functions occurring in our adult *C. daubneyi* specimens, the predicted peptide sequences generated through Transdecoder (https://transdecoder.github.io) within the Trinotate annotation process were extracted for the top 10% of mapped contigs (7379) and uploaded to the BlastKOALA annotation tool [26] for K number assignment of sequences categorized according to the KEGG Orthology system (ko00001).

Protein families representing members of the Phase I, II and III detoxification pathways were investigated within the *C. daubneyi* transcriptome. Sequences from members of the cytochrome P450 (CYP450) family identified in Cwiklinski et al. [27], the glutathione transferase (GST) from Morphew et al. [28] and the Fatty Acid Biding Protein (FABP) family from Morphew et al. [29] were used to BLAST the *C. daubneyi* transcriptome. The E value for BLAST analysis was set at 1. All transcript BLAST hits were initially confirmed as CYP450s, GSTs or FABPs using BLAST analysis against the GenBank database. In addition, sequences were analysed for Interpro domains [30] specific to CYP450 (IPR023173 NADPH-cytochrome P450 reductase or IPR036396 cytochrome P450 superfamily for reductases and monooxygenases), GST (IPR036282 glutathione S-transferase, C-terminal domain superfamily and/or IPR004045 glutathione S-transferase, N-terminal) and FABP (minimum inclusion of IPR012674 Calycin supported with IPR031259 intracellular lipid binding protein, IPR000566 lipocalin/cytosolic fatty-acid binding domain and IPR000463 cytosolic fatty-acid binding). Furthermore, FABPs were classified as FABPs using secondary structure prediction using PsiPred [31] looking for the characteristic 2 alpha helices and 10 beta sheets.

### Proteomics: sample collection

Samples of rumen fluke parasites from naturally infected cattle were obtained from a local abattoir (mid-Wales, UK). Flukes were washed in warm (39 °C) phosphate-buffered saline (PBS) to remove rumen content contamination on collection, and transported directly to the laboratory. The PBS solution was replaced with a fresh volume for a further 10 min wash on arrival at the laboratory. Live parasites were then transferred into warm (39 °C) DME culture media (DMEM) supplemented with 15 mM HEPES, 61 mM glucose, 2.2 mM calcium acetate, 2.7 mM magnesium sulphate, 1 μM serotonin and gentamycin (5 μg/ml) as described previously [32], allowing 1 ml of culture media per fluke. After a 6-hour culture period, parasites were removed from the culture liquid and both parasites and liquid were snap frozen in liquid nitrogen prior to storage at -80 °C.

### Protein sample isolation and 2D SDS-PAGE

Excretory/secretory (ES) protein samples: a protease inhibitor cocktail (cOmplete™ tablet, Roche, Welwyn Garden City, UK) was added to the liquid samples before being clarified by centrifugation at 45,000× *g* for 45 min at 4 °C. The resulting supernatant was then concentrated using an Amicon® 400 ml stirred cell unit and an Ultracel® 10 kDa MWCO regenerated cellulose ultrafiltration membrane disc (Merck Millipore, Darmstadt, Germany). Concentrated ES proteins were precipitated using an equal volume of ice cold 20% v/v TCA in acetone. Precipitated protein pellets were washed twice in ice cold acetone, dried at -20 °C before solubilisation in buffer as described by Morphew et al. [32] Somatic soluble protein samples: whole parasite samples were homogenised in buffer containing 20 mM potassium phosphate (pH 7.4), 0.1% v/v Triton X 100 and a protease inhibitor cocktail tablet (Roche mini cOmplete™). Soluble protein samples were clarified by centrifugation at 100,000× *g* for 45 min at 4 °C. Proteins were then precipitated and re-solubilised from the supernatant as for ES products.

Protein concentration was measured by the Bradford assay [33] and 17 cm immobilised pH gradient IPG strips (Bio-Rad, Watford, UK) were rehydrated with a total of 250 and 500 μg of protein for the ES and somatic samples respectively. A total sample volume of 300 μl was used to rehydrate and focus the 17 cm pH 3–10 IPG strips (Bio-Rad) at 20 °C for separation in the first dimension. Linear IPG strips were used for somatic samples with non-linear IPG strips used for improved resolution of protein spots with the ES samples. IPG strips were focussed to between 60,000 and 80,000 Vh using the Protean IEF Cell (Bio-Rad). Each IPG strip was then equilibrated for 15 min in equilibration buffer [containing 50 mM Tris-HCl pH 8.8, 6 M urea, 30% glycerol (v/v) and 2% SDS (w/v)) with the addition of DTT (Melford, UK) at 10 mg/ml] followed by a second equilibration with IAA (Sigma-Aldrich, Gillingham, UK) at 25 mg/ml replacing DTT [34]. The IPG strips were separated in the second dimension on the Protean II system (Biorad) using 14% polyacrylamide gels as described by Morphew et al. [32]. Gels were then fixed in 40 % (v/v) ethanol, 10 % (v/v) acetic acid and stained using Colloidal Coomassie [35].

### Imaging and spot identification

Coomassie stained gels were imaged using a GS-800 calibrated densitometer (Bio-Rad) set for coomassie stained gels at 400 dpi. Gel images were analysed using Progenesis PG220 v.2006 using the ‘Mode of non-spot’ background subtraction method. Average gels were created from 4 replicate gels for the somatic and ES samples respectively and normalised spot volumes were calculated using the ‘Total spot volume multiplied by total area’ method to determine the most abundant protein spots.

### Mass spectrometry and data analysis

The 50 most abundant spots were identified for both ES and somatic samples on 17 cm SDS-PAGE gels using Progenesis and excised before being subjected to tryptic digest [34]. Digested protein samples were resuspended in 20 μl 0.1% formic acid for LC Tandem mass spectrometry (MS/MS) analysis on an Agilent 6550 iFunnel Q-TOF mass spectrometer with a Dual AJS ESI source coupled to a 1290 series HPLC system (Agilent, Cheshire, UK). A 2.1 × 50 mm 1.8 micron Zorbax Eclipse Plus C18 column was used; 10 μl of sample was injected for analysis. Liquid chromatography was performed at a flow of 0.1 ml/min with a piece-linear gradient using water with 0.1% v/v formic acid (A) and acetonitrile with 0.1% v/v formic acid (B) (0–3% B over 2 min, 3–40% B over 7 min, 40–100% B over 1 min, hold at 100% B for 1 min).

Ions were generated using a Dual AJS ESI source. MS/MS was performed in Auto MS/MS mode in the 300–1700 range, at a rate of 0.6 spectra per second, performing MS2 on the 5 most intense ions in the precursor scan. Masses were excluded for 0.1 min after MS2 was performed. Reference mass locking was used for internal calibration using the mass of 922.009798 Da. Peak lists were generated using Mass Hunter Qualitative Analysis software (version B.06.00) using Molecular Feature Extraction and exported as Mascot Generic Files. MSMS data was analysed with MASCOT (Version 2.4.1; www.matrixscience.com) using an MS/MS Ions search on standard settings (precursor tolerance ± 1.2 Da, fragment ion tolerance ± 0.6 Da) for the enzyme trypsin, allowing up to 2 missed cleavages, carbamidomethyl as a fixed modification and oxidation of methionine as a variable modification. Spectra were searched against the in-house transcript assembly for *C. daubneyi* described in the present study, with sequence hits reported from MASCOT compared to the functionally annotated transcript data for protein ID. The mass spectrometry proteomics data have been deposited to the ProteomeXchange Consortium *via* the PRIDE partner repository with the dataset identifier PXD007772 and null. UniProt identifiers from the transcript BLASTp annotation data which matched to each protein spot following LC MS/MS were then uploaded to the UniProt mapping application (uniprot.org/mapping, accessed 03/12/2015) to obtain descriptive data on the protein matches, organism ID and GOslim information for each matched protein. SignalP and TMHMM matches for ES proteins, along with gene component expression levels as FPKM values were extracted from the transcript annotation data to match each hit. Evidence of the 50 most abundant proteins being identified as packaged in exosome-like vesicles in previous studies was identified by matching protein descriptions to those obtained in previous helminth exosome studies [36–39] or in the ExoCarta database [40].

### Extracellular-like vesicle visualisation

Isolation of extracellular-like vesicles was performed by ultracentrifugation of ES products (700× *g* for 20 min at 4 °C), followed by 120,000× *g* for 80 min at 4 °C, using a Optima™ L-100 XP ultracentrifuge (Beckman Coulter, High Wycombe, UK) using a Type 70 Ti rotor as described by Nowacki et al. [39] with the addition of a 2.0 μm syringe filter step before the final pelleting and re-suspension to eliminate contaminating bacterial components which may have been present from the rumen. Imaging and identification of extracellular vesicles using a Jeol 1010 transmission electron microscope (TEM) and size-selective criteria (30–100 μM) was performed as previously described [39].

### *In vitro* gas production culture

Liquid fraction rumen fluid for the *in vitro* culture protocol was collected from 5 individual bovine rumens, each visually inspected and declared free from rumen fluke infection before fluid collection. Rumen fluid was collected immediately *post-mortem* after inspection upon opening of the rumens by straining the rumen contents through a layer of muslin cloth into a pre-warmed thermos collection flask. Flasks were filled almost to the brim to minimise the headspace for oxygenation of the rumen fluid. In total, 40 250 ml Duran™ bottles (ThermoFisher Scientific, Paisley, UK) were used as the culture vessels. Half of these bottles were supplied with 1 g dried and ground grass silage as a fermentation substrate for microbial activity. Half of the bottles were left empty. These groups were then split, with half of the silage containing and half of the empty bottles to receive rumen fluke and half without in order to provide both positive and negative controls for the fermentation. A single replicate bottle for each of the 5 animals sampled for rumen fluid was utilised in each treatment and treatments were replicated at 2 different time points; 6 and 24 hours. Culture treatment groups were therefore as follows: Group 1 (Blank; no rumen fluke and no silage), Group 2 (Fluke only; rumen fluke but no silage), Group 3 (Rumen fluke and Silage) and Group 4 (Silage only). On return to the laboratory, rumen fluid from each of the 5 individual animals was mixed 1:1 with pre-prepared Coleman-Simplex buffer based on the medium described by Coleman [41]. This mixture was maintained in a 39 °C water bath and continuously flushed with CO_2_ to maintain anaerobic conditions during dispensing. To each culture bottle, 100 ml of the 50% rumen fluid mixture was added and for fluke-positive bottles 10 rumen fluke were added. Bottles were sealed using ANKOM RF Gas Production Measurement System units to record cumulative gas production and allow for automated pressure release. Based on the number of parasites present in a high burden rumen fluke infection detected in a previous slaughterhouse study (11,895 in a single bovine) [42] and an estimated rumen volume of 100 l, 10 parasites were added to the 100 ml culture vessels in order to simulate a high *in vivo* burden.

### Gas production analysis

The volumes of gas produced were measured using an ANKOM RF Gas Production Measurement System (Macedon, NY, USA), with data collected every 5 min from each bottle over a 24 h period. Cumulative gas production levels obtained from the ANKOM RF Gas Production system were fitted to the exponential equation using the Neway Excel curve-fitting program, Fit Curve [43] (Obtained from http://www.macaulay.ac.uk/IFRU/resrc_fcurve.html, April 2016) as described by Ørskov & McDonald [44].

### Metabolite analysis

VFA analysis was performed using 4 ml of sample from each bottle and mixed with 1 ml of 20% v/v orthophosphoric acid containing 4 mM 2-ethyl butyric acid (internal standard). During storage, VFA samples had settled to provide a clear supernatant within each 15 ml falcon tube, 2 ml of this was syringe filtered through a 0.45 μm nylon syringe filter tip (ChronusFilter, SMI-LabHut Ltd, Gloucester, UK) and transferred into a GC vial (Chromacol, Altrincham, UK). Vials were analysed using Gas Chromatography on a Varian CP-3380 GC instrument with a HP-FFAP 25 m × 0.53 mm I.D. × 1 μm film thickness column (J and W Scientific, USA). Data collection and analysis was carried out using the Varian Galaxie Chromatography Workstation (software version 1.9.3.2.) to calculate the mmol/l concentration of different VFAs within each vial. Data was then imported into Microsoft Excel for calculation of the mmol/l concentration of VFAs within the *in vitro* rumen fermentation samples.

Stored culture samples were defrosted overnight at 4 °C. Ammonia levels were determined from the contents of each fermentation bottle. Each sample was put into 10% [w/v] TCA and centrifuged for 15 min at 14,000× *g* at 4 °C. In preparation for analysis the sample was diluted in deionised water 5-fold. A sample of this dilution was mixed with Reagent A (13 mg/l NaOH, 4 mg/l EDTA), Reagent B (10 g/l phenol, 50 μg/l sodium nitroprusside) and Reagent C (5 g/l of NaOH in 15% (v/v) sodium hypochlorite). Reactions were then performed for 15 min in the dark at 39 °C before reading the absorbance at 630 nm.

### Protozoan counts

Samples of the rumen culture liquid were diluted 1:1 in 0.9% (w/v) NaCl, 4% (v/v) formalin to preserve the protozoans. A minimum of 24 h before counting, methylene blue dye was added to stain the protozoans. Samples were then diluted 1:20 in NaCl/formalin and mixed by pipetting before 10 μl of this dilution was placed on a microscope slide under a cover slip for examination using the 20× magnification objective lens on a bright field light microscope. All visible protozoans in the 10 μl volume placed under the cover slip were counted. Samples from each fermentation bottle were counted in duplicate and an average count, per 10 μl, calculated. Average counts were then corrected for the dilution factors used during sample preparation and the total counts of protozoans present within each fermentation bottle analysed by one-way ANOVA for both the 6 and 24 h time points as described below for the 5 replicates.

### Statistical analysis

GenStat software (16th edition, VSN International, UK) was used to perform a one-way ANOVA test with Bonferroni corrections for *post-hoc* analyses, for each variable measured at each respective time point to detect differences occurring from either fluke or silage treatments of fermentation vessels. For each ANOVA, animal (the 5 donor animals from which rumen fluid was sampled for the fermentations) was used as a blocking factor in order to account for individual variation in the rumen fluid sample pools.

### Analysis of propionate production pathway genes present in the *C. daubneyi* transcriptome

Known genes from the related trematode species, for which data exists in the KEGG database, *S. mansoni*, along with data for the nematode *C. elegans* as the best annotated of the 5 nematode species for which data was available in KEGG were used to identify the *C. daubneyi* propionate pathway. Species-specific maps of the propionate production pathway (map 00640) were viewed via the KEGG pathway application and for each enzyme code indicated, as identified in the respective data for *S. mansoni* and *C. elegans*, peptide sequences were downloaded and a local tBLASTn search performed using BioEdit [45] against the mapped *C. daubneyi* transcript contigs with a stringent E-value score of 1.0 × 10^-80^ applied to reported hits. Using the user data mapping application within KEGG pathway maps enzyme codes for which actively transcribed genes were identified were highlighted on the propionate metabolism map.

## Results

### Transcriptome analysis

This Transcriptome Shotgun Assembly project has been deposited at DDBJ/EMBL/GenBank under the accession GFUT00000000. The version described in this paper is the first version, GFUT01000000. From a total of 226,188,786 raw reads, the Trinity transcript assembly generated 103,541 unique contigs (Table 1). Of the contigs generated during the Trinity assembly process, 73,792 mapped back to the raw Illumina sequencing reads. Only these mapped reads were included in downstream analysis. From these 73,792 unique contigs, 54,617 individual gene components (defined in Trinotate as genes and their associated duplicates, gene parts and fragments in the absence of a reference genome) were identified of which 69.51% had no annotation associated with them after functional analysis using the Trinotate pipeline. Gene components were sorted by cumulative FPKM values for all isoforms identified and sequences of the 50 most highly expressed gene components (longest isoform shown) were identified (Additional file 1: Table S1). Of these gene components, 37 had a top annotation hit to another helminth species, 9 had no successful annotation and 4 hit to other organisms (ciliate protozoa (2), zebrafish and a yeast). These most highly expressed genes were largely annotated as eggshell and vitelline proteins, required for reproduction, followed by associations with respiratory processes, tubulin, ferritin and a number of unannotated or uncharacterised transcripts were identified (Additional file 1: Table S1). A full list of the identified transcripts is provided in Additional file 1: Table S2.

**Table 1:** *De novo* transcriptome assembly summary statistics for adult *C. daubneyi* specimens collected from a natural bovine infection following RNAseq and Trinity assembly

Category	Statistic
Total raw reads	226,188,786
Per replicate	
A	95,417,496
B	74,644,572
C	56,126,718
GC percentage	47
Total assembled contigs generated	103,541
Contigs mapped to raw reads	73,792
Average contig length (bp) (mapped)	738 (892)
Maximum contig length (bp)	24,404
Minimum contig length (bp)	224
% mapped contigs without BLASTx/p annotation	62.94/62.74
% Mapped contigs with Signal Peptide (SigP)	1.80
% Mapped contigs with Transmembrane Domain (TM)	5.77
% Mapped contigs with both TM+SigP	0.65
Unique gene components identified (from mapped contigs)	54,617
% gene components without BLASTx/p annotation	69.51

The 50 gene components with the highest FPKM values that had no annotation data from the Trinotate analysis were additionally searched against the NCBInr database (http://blast.ncbi.nlm.nih.gov/Blast.cgi, accessed 14/12/2015) and the SRA files generated from Ion Torrent™ sequencing of the transcriptome of the related paramphistome *P. cervi* [25] [available under the accession numbers SRA091604 (sheep), SRA039814 (goat) and SRA091607 (buffalo): Additional file 1: Table S3]. Only 1 additional significant hit was returned from the NCBInr database search, matching gene component TR26203|c0_g1 to a *C. daubneyi cox*1 gene with 100% homology (8% coverage) to accession number KJ574061.1. BLAST searches against the SRA files for *P. cervi* found significant matches for 38 of the 50 gene components searched, suggesting that these unidentified gene components may be common across the paramphistomes and the 12/50 unmatched gene components are potentially unique to *C. daubneyi*.

**Fig. 1:**
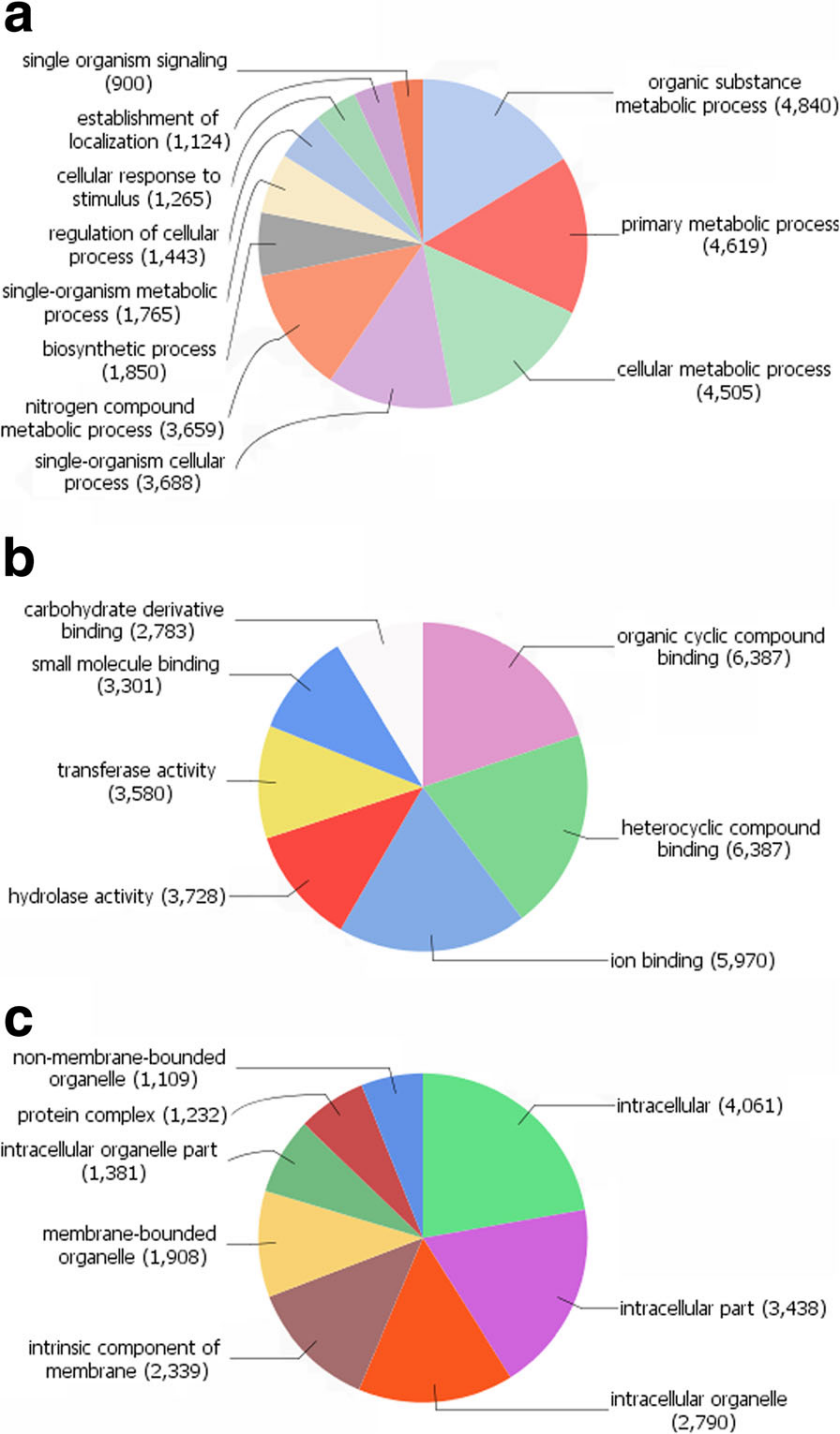
Annotation of mapped *C. daubneyi* sequences to Gene Ontology (GO) terms at GO level 3. Numbers of sequences assigned to each term are shown in parentheses with a total of 17,149 sequences returned at least 1 (range 1–15) GO term annotation. **a** Biological process. **b** Molecular function. **c** Cellular component

In total, 17,149 sequences were matched to GO terms (level 3) during the Trinotate annotation process (Fig. 1). At level 3, the majority of GO terms identified under the category Biological Process were related to organic substance, primary and cellular metabolic processes and single-organism processes. Terms identified for Molecular Functions were mostly linked to binding activities and Cellular Component terms were in the majority related to intracellular and membrane component terms. Categorisation of the predicted peptide sequences corresponding to the top 10% of expressed contigs using the BlastKOALA tool and KEGG KO orthology system showed that highly expressed contigs were most commonly linked to genes within the KEGG database associated with genetic information processing, cellular processes, human diseases (not including parasitic infections) and environmental information processing. Of the peptide sequences uploaded, only 28.7% could be annotated to KO numbers in the KEGG database.

In summary, sequences for which an annotation could be obtained, matches were mostly common to related trematode, cestode or nematode species as would be expected. The transcriptome assembly produced mapped reads of which 62.94% appeared to be potentially novel sequences with no matches identified during the Trinotate (V2.0) functional annotation process, highlighting the dearth of current data available in relation to the paramphistomes and that these parasites may be quite unique amongst the trematodes. This was further exemplified by the attainment of only 28.7% annotation of predicted peptides corresponding to the 10% most expressed contigs submitted through BlastKOALA for annotation to functional orthologous groups in the KEGG database.

As a case study, phase I, II and III detoxification components were investigated given the likely role they play in both anthelmintic detoxification and detoxification for survival within the rumen environment. Phase I, II and III target families, namely CYP450s, GSTs and FABPs respectively, were all represented in the *C. dauvbneyi* transcriptome. Analysis of the phase I CYP450s identified one gene product corresponding to a CYP450 monooxygenase and one CYP450 reductase. Both were confirmed as such with key motifs for the monooxygenase (IPR036396 cytochrome P450 superfamily) and reductase (IPR023173 NADPH-cytochrome P450 reductase). Phase II detoxification demonstrated an increase in sequences identified from BLAST analysis. A total of 19 GST gene products were identified representing 4 established GST classes. Two gene products represented Omega class GSTs whilst a single gene product was confirmed as a zeta class GST. Of the more abundant platyhelminth classes 2 mu class GSTs were identified along with a further 14 sigma class like GST gene products. All GST sequences were confirmed as GSTs using the Interpro domain prediction of IPR036282. Finally, investigating the phase III sequestration FABPs revealed a total of 17 full length FABPs along with 2 further partial sequences representing 19 potential FABPs. Of note are FABP gene products representing the *Fasciola* type V FABPs and a single representative for *Fasciola* type III, IV and VII (Additional file 2: Figure S1). Of interest is the expansion of a two groups of *C. daubneyi* FABPs, containing two and ten FABP isoforms designated CdFABP IL 1 and CdFABP IL2 respectively, that align more to vertebrate ileal and liver FABPs than they do Fasciolids or other platyhelminths.

### Proteomic profiles

**Fig. 2:**
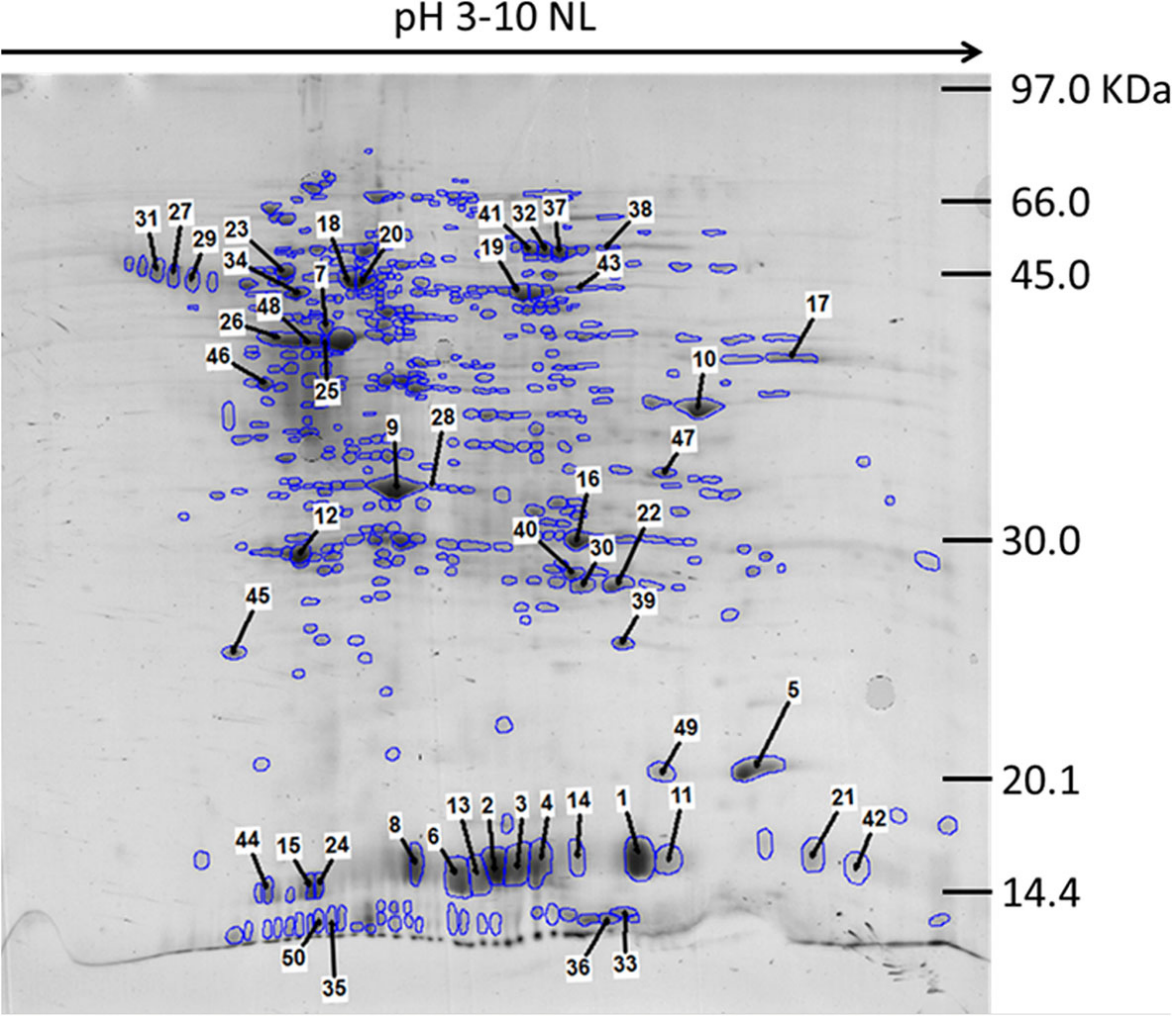
Representative 2DE protein array of adult *C. daubneyi* excretory-secretory (ES) products. 17 cm 2DE protein array of excretory-secretory (ES) products from *in vitro* culture, annotated to highlight the 50 most abundant spots identified during Progenesis analysis. Proteins were separated across a non-linear pH range of 3–10 using IEF in the first dimension and 14% SDS-PAGE in the second dimension and Coomassie Blue stained. ES products were obtained from 6 h *in vitro* culture in supplemented DME medium. Numbered protein spots correspond to the order of relative abundance detected by Progenesis analysis and show the spots excised for MS identification

**Fig. 3:**
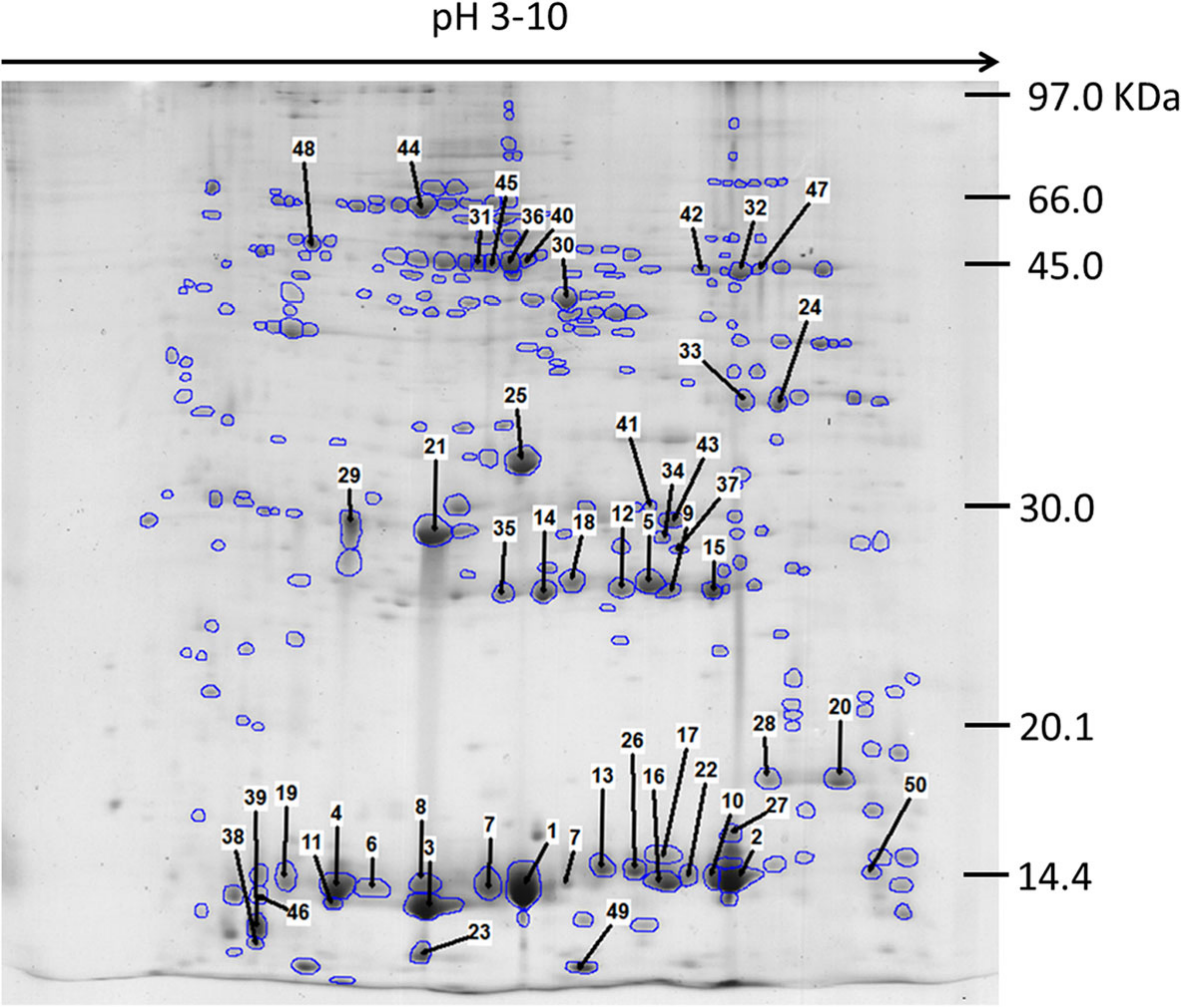
Representative 2DE protein array of adult *C. daubneyi* soluble somatic proteins. 17 cm 2DE protein array of soluble somatic proteins annotated to highlight the 50 most abundant spots identified during Progenesis analysis. Proteins were separated across a linear pH range of 3–10 using IEF in the first dimension and 14% SDS-PAGE in the second dimension and Coomassie Blue stained. Numbered protein spots correspond to the order of relative abundance detected by Progenesis analysis and show the spots excised for MS identification

Both *in vitro* cultured ES and somatic proteome samples produced consistent 2D gel profiles allowing high quality Progenesis average gels to be generated (Figs. 2 and 3, respectively), with the most abundant 50 spots from each average gel identified. These spots were then excised and subjected to tryptic digest and LC MS/MS, with the resulting peak spectra searched against our new functionally annotated *C. daubneyi* transcript database for protein identification, with putative spot identifications in Tables 2 and 3 for ES and somatic samples, respectively (full details are provided in and detailed further in Additional file 1: Tables S4 and S5). Only 1 spot from the somatic profile (spot 50) failed to return any hit and in total 6 spots (numbers 7, 15, 18, 20, 28 and 45) in the ES profile only returned hits with a MASCOT score below the significance threshold of 49. The most highly abundant spots in the ES profile were identified as uncharacterised proteins belonging to the calycin superfamily/fatty acid binding protein family and represent CdFABP III (TR17138), CdFABP IL1 (TR14337) and CdFABP IL2 (TR18162). Identified proteins also included peptidases and proteases (including cathepsins) and glutathione transferase (GST) proteins which are known for their role in detoxification and protein-protein interactions. Of note were the identification of GSTs representing both mu and sigma class GSTs in the ES products but limited to one representative of both (isoforms of TR17112 mu class and TR21279 sigma-like). In the somatic profile uncharacterised proteins were again common and largely identified as fatty acid binding protein (FABP) family proteins representing CdFABP III (TR17138), CdFABP IL1 (TR15960) and CdFABP IL2 (TR18162). GSTs were also abundant with representatives from both mu and sigma-like GST classes characterised by TR17112 and TR21279 isoforms respectively mirroring that of the ES profile abundant GSTs. In addition, dehydrogenases/reductases and globins featured prominently in the somatic proteome.

**Table 2:** Top 50 most abundant proteins from the excretory/secretory proteome profile of adult *C. daubneyi*. Putative proteins were identified by 2D SDS-PAGE and LC MS/MS. The most significant match is shown for each protein spot scoring above the MASCOT score significance threshold of 47 (spot numbers designated with an * indicate the best match was below the threshold). Putative identifications in bold correspond to gene components identified in the top 50 from the transcript analysis. Previous identification in extracellular-like vesicles is indicated if the same or a similar protein has been reported in parasite extracellular-like vesicles [35–38] or in the ExoCarta database [39]

Spot	Gene ID	Isoform	MASCOT Score	Uniref90 BLASTp annotation	Alternative annotation	UniProt description	Protein superfamily	Organism	Signal peptide	TM domain	ID in exosomes
1	**TR17138|c0_g1**	**i1**	**60**	**A0A074ZQZ2_9TREM**	**–**	**Uncharacterized protein**	**Calycin superfamily; fatty-acid binding protein (FABP) family**	***Opisthorchis viverrini***	**0**	**0**	**–**
2	**TR18162|c0_g1**	**i1**	**255**	**A0A075AK05_9TREM**	**–**	**Uncharacterized protein**	**Calycin superfamily; fatty-acid binding protein (FABP) family**	***Opisthorchis viverrini***	**0**	**0**	**–**
3	**TR18162|c0_g1**	**i1**	**177**	**A0A075AK05_9TREM**	**–**	**Uncharacterized protein**	**Calycin superfamily; fatty-acid binding protein (FABP) family**	***Opisthorchis viverrini***	**0**	**0**	**–**
4	**TR18162|c0_g1**	**i1**	**199**	**A0A075AK05_9TREM**	**–**	**Uncharacterized protein**	**Calycin superfamily; fatty-acid binding protein (FABP) family**	***Opisthorchis viverrini***	**0**	**0**	**–**
5	**TR17982|c0_g1**	**i1**	**620**	**I2K1A3_DEKBR**	**–**	**Peptidyl-prolyl cis-trans isomerase (EC 5.2.1.8)**	**Cyclophilin-type PPIase family**	***Brettanomyces bruxellensis*** **AWRI1499**	**0**	**0**	**Yes**
6	**TR18162|c0_g1**	**i1**	**280**	**A0A075AK05_9TREM**	**–**	**Uncharacterized protein**	**Calycin superfamily; fatty-acid binding protein (FABP) family**	***Opisthorchis viverrini***	**0**	**0**	**–**
7*	TR31531|c0_g1	i1	27	0	No annotations	na	na	na	0	0	**–**
8	**TR18162|c0_g1**	**i5**	**343**	**A0A075AK05_9TREM**	**–**	**Uncharacterized protein**	**Calycin superfamily; fatty-acid binding protein (FABP) family**	***Opisthorchis viverrini***	**0**	**0**	**–**
9	TR17318|c0_g1	i1	52	Q5DDT1_SCHJA	**–**	Putative vesicle-associated membrane protein-associated protein A (SJCHGC09425 protein)	0	*Schistosoma japonicum* (blood fluke)	0	Yes	Yes
10	TR17219|c0_g1	i1	590	G4V7Z7_SCHMA	**–**	Malate dehydrogenase (EC 1.1.1.37)	LDH/MDH superfamily, MDH type 2 family	*Schistosoma mansoni* (blood fluke)	0	0	Yes
11	**TR17138|c0_g1**	**i1**	**141**	**A0A074ZQZ2_9TREM**	**–**	**Uncharacterized protein**	**Calycin superfamily; fatty-acid binding protein (FABP) family**	***Opisthorchis viverrini***	**0**	**0**	
12	TR11982|c0_g1	i1	221	G4LUJ6_SCHMA	**–**	Proteasome subunit beta type (EC 3.4.25.1)	Peptidase T1B family	*Schistosoma mansoni* (blood fluke)	0	0	Yes
13	**TR18162|c0_g1**	**i1**	**226**	**A0A075AK05_9TREM**	**–**	**Uncharacterized protein**	**Calycin superfamily; fatty-acid binding protein (FABP) family**	***Opisthorchis viverrini***	**0**	**0**	**–**
14	**TR17138|c0_g1**	**i1**	**97**	**A0A074ZQZ2_9TREM**	**–**	**Uncharacterized protein**	**Calycin superfamily; fatty-acid binding protein (FABP) family**	***Opisthorchis viverrini***	**0**	**0**	**–**
15*	TR14337|c1_g3	i2	44	0	FABP1_FASGI	Fatty acid-binding protein 1	Calycin superfamily; fatty-acid binding protein (FABP) family	*Fasciola gigantica* (giant liver fluke)	0	0	Yes
16	TR18374|c0_g1	i1	663	S4UI50_FASHE	**–**	Triosephosphate isomerase (EC 5.3.1.1)	Triosephosphate isomerase family	*Fasciola hepatica* (liver fluke)	0	0	Yes
17	**TR22034|c1_g4**	**i3**	**761**	**A0A075A0M6_9TREM**	**–**	**Fructose-bisphosphate aldolase (EC 4.1.2.13)**	**Class I fructose-bisphosphate aldolase family**	***Opisthorchis viverrini***	**0**	**0**	**Yes**
18*	TR31531|c0_g1	i1	26	0	No annotations	na	na	na	0	0	**–**
19	TR17367|c0_g1	i1	642	G7YF68_CLOSI	**–**	Enolase	0	*Clonorchis sinensis* (Chinese liver fluke)	0	0	Yes
20*	TR15346|c0_g1	i1	33	G4VM15_SCHMA	**–**	Putative eukaryotic translation initiation factor 4b/4h	0	*Schistosoma mansoni* (blood fluke)	0	0	**–**
21	**TR18162|c0_g1**	**i1**	**118**	**A0A075AK05_9TREM**	**–**	**Uncharacterized protein**	**Calycin superfamily; fatty-acid binding protein (FABP) family**	***Opisthorchis viverrini***	**0**	**0**	**–**
22	TR21279|c0_g5	i1	202	C1LY00_SCHJA	**–**	Glutathione S-transferase (EC 2.5.1.18)	0	*Schistosoma japonicum* (blood fluke)	0	0	Yes
23*	TR22414|c0_g1	i1	27	0	No annotations	na	na	na	0	0	**–**
24	**TR18162|c0_g1**	**i1**	**124**	**A0A075AK05_9TREM**	**–**	**Uncharacterized protein**	**Calycin superfamily; fatty-acid binding protein (FABP) family**	***Opisthorchis viverrini***	**0**	**0**	**–**
25	TR43052|c0_g1	i1	53	0	No annotations	na	na	na	0	0	**–**
26	TR18535|c1_g1	i1	75	U6P7F0_HAECO	**–**	Uncharacterized protein	Peptidase C1 family	*Haemonchus contortus* (Barber pole worm)	0	0	**–**
27	TR20917|c0_g1	i1	230	Q4VRW5_9TREM	**–**	Cathepsin B1 isotype 5	Peptidase C1 family	*Trichobilharzia regenti*	Yes	0	Yes
28*	TR23151|c0_g1	i1	29	G7YVU0_CLOSI	**–**	ADIPOR-like receptor CG5315	0	*Clonorchis sinensis* (Chinese liver fluke)	0	Yes	**–**
29	TR20917|c0_g1	i1	365	Q4VRW5_9TREM	**–**	Cathepsin B1 isotype 5	Peptidase C1 family	*Trichobilharzia regenti*	Yes	0	Yes
30	TR21279|c0_g5	i1	142	C1LY00_SCHJA	**–**	Glutathione S-transferase (EC 2.5.1.18)	0	*Schistosoma japonicum* (blood fluke)	0	0	Yes
31	TR20917|c0_g1	i1	255	Q4VRW5_9TREM	**–**	Cathepsin B1 isotype 5	Peptidase C1 family	*Trichobilharzia regenti*	Yes	0	Yes
32	TR23254|c0_g1	i1	595	C6FWH0_CLOSI	**–**	Leucine aminopeptidase 2 (Leucyl aminopeptidase)	0	*Clonorchis sinensis* (Chinese liver fluke)	0	0	Yes
33	TR13284|c0_g1	i1	74	F6YCC0_HORSE	**–**	Uncharacterized protein	0	*Equus caballus* (horse)	Yes	0	**–**
34	TR10146|c0_g1	i1	49	0	No annotations	na	na	na	0	0	**–**
35	TR16703|c0_g1	i1	89	J9HU03_9SPIT	**–**	Cystatin B, putative	0	*Oxytricha trifallax*	0	Yes	Yes
36	TR13284|c0_g1	i1	112	F6YCC0_HORSE	**–**	Uncharacterized protein	0	*Equus caballus* (horse)	Yes	0	**–**
37	TR23254|c0_g1	i1	576	C6FWH0_CLOSI	**–**	Leucine aminopeptidase 2 (Leucyl aminopeptidase)	0	*Clonorchis sinensis* (Chinese liver fluke)	0	0	Yes
38	TR23254|c0_g1	i1	309	C6FWH0_CLOSI	**–**	Leucine aminopeptidase 2 (Leucyl aminopeptidase)	0	*Clonorchis sinensis* (Chinese liver fluke)	0	0	Yes
39	TR18926|c0_g1	i1	137	V4B1M6_LOTGI	**–**	Peptidyl-prolyl cis-trans isomerase (EC 5.2.1.8)	Cyclophilin-type PPIase family	*Lottia gigantea* (giant owl limpet)	0	Yes	Yes
40	TR17112|c0_g1	i1	299	Q25595_CLOSI	**–**	Putative glutathione transferase	GST superfamily	*Clonorchis sinensis* (Chinese liver fluke)	0	0	Yes
41	TR23254|c0_g1	i1	613	C6FWH0_CLOSI	**–**	Leucine aminopeptidase 2 (Leucyl aminopeptidase)	0	*Clonorchis sinensis* (Chinese liver fluke)	0	0	Yes
42	**TR18162|c0_g1**	**i1**	**105**	**A0A075AK05_9TREM**	**–**	**Uncharacterized protein**	**Calycin superfamily; fatty-acid binding protein (FABP) family**	***Opisthorchis viverrini***	**0**	**0**	**–**
43	TR17367|c0_g1	i1	438	G7YF68_CLOSI	**–**	Enolase	0	*Clonorchis sinensis* (Chinese liver fluke)	0	0	Yes
44	**TR18162|c0_g1**	**i1**	**84**	**A0A075AK05_9TREM**	**–**	**Uncharacterized protein**	**Calycin superfamily; fatty-acid binding protein (FABP) family**	***Opisthorchis viverrini***	**0**	**0**	**–**
45*	TR21653|c0_g1	i1	30	G4VA64_SCHMA	**–**	Palmitoyltransferase (EC 2.3.1.225)	DHHC palmitoyltransferase family	*Schistosoma mansoni* (blood fluke)	0	Yes	**–**
46	TR25811|c0_g1	i1	79	0	A0A085M8I9_9BILA	Uncharacterized protein	0	*Trichuris suis* (pig whipworm)	0	0	**–**
47	**TR20361|c12_g1**	**i1**	**294**	**H2KV75_CLOSI**	**–**	**Putative eggshell protein**	**0**	***Clonorchis sinensis*** **(Chinese liver fluke)**	**Yes**	**Yes**	**–**
48	TR18535|c1_g1	i1	76	U6P7F0_HAECO	**–**	Uncharacterized protein	Peptidase C1 family	*Haemonchus contortus* (Barber pole worm)	0	0	–
49	**TR17982|c0_g1**	**i1**	**519**	**I2K1A3_DEKBR**	**–**	**Peptidyl-prolyl cis-trans isomerase (EC 5.2.1.8)**	**Cyclophilin-type PPIase family**	***Brettanomyces bruxellensis*** **AWRI1499**	**0**	**0**	**Yes**
50	TR16703|c0_g1	i1	54	J9HU03_9SPIT	**–**	Cystatin B, putative	0	*Oxytricha trifallax*	0	Yes	Yes

*Abbreviation*: *na* not applicable

**Table 3:** Top 50 most abundant proteins from the soluble somatic proteome profile of adult *C. daubneyi*. Putative proteins were identified by 2D SDS-PAGE and LC MS/MS. The most significant match is shown for each protein spot scoring above the MASCOT score significance threshold of 47 (spot numbers designated with an * indicate the best match was below the threshold). Putative identifications in bold correspond to gene components identified in the top 50 from the transcript analysis

Spot	Gene ID	Isoform	MASCOT score	Uniref90 BLASTp annotation	Alternative annotation	UniProt description	Organism	Protein family
**1**	TR14423|c0_g1	i1	226	T1K7W8_TETUR	–	Uncharacterized protein	*Tetranychus urticae* (two-spotted spider mite)	0
**2**	**TR17138|c0_g1**	**i1**	**244**	**A0A074ZQZ2_9TREM**	–	**Uncharacterized protein**	***Opisthorchis viverrini***	**Calycin superfamily; fatty-acid binding protein (FABP) family**
**3**	**TR18162|c0_g1**	**i1**	**391**	**A0A075AK05_9TREM**	–	**Uncharacterized protein**	***Opisthorchis viverrini***	**Calycin superfamily; fatty-acid binding protein (FABP) family**
**4**	**TR18162|c0_g1**	**i1**	**288**	**A0A075AK05_9TREM**	–	**Uncharacterized protein**	***Opisthorchis viverrini***	**Calycin superfamily; fatty-acid binding protein (FABP) family**
**5**	TR17112|c0_g1	i1	193	Q25595_CLOSI	–	Putative glutathione transferase	*Clonorchis sinensis* (Chinese liver fluke)	GST superfamily
**6**	**TR18162|c0_g1**	**i1**	**158**	**A0A075AK05_9TREM**	–	**Uncharacterized protein**	***Opisthorchis viverrini***	**Calycin superfamily; fatty-acid binding protein (FABP) family**
**7**	TR17170|c0_g1	i1	193	0	No annotations	na	na	na
**8**	**TR18162|c0_g1**	**i1**	**247**	**A0A075AK05_9TREM**	–	**Uncharacterized protein**	***Opisthorchis viverrini***	**Calycin superfamily; fatty-acid binding protein (FABP) family**
**9**	TR21279|c0_g5	i1	150	C1LY00_SCHJA	–	Glutathione S-transferase (EC 2.5.1.18)	*Schistosoma japonicum* (blood fluke)	0
**10**	TR18466|c1_g2	i1	300	Q5D2M7_9TREM	–	Myoglobin 1	*Paragonimus westermani*	Globin family
**11**	**TR18162|c0_g1**	**i1**	**220**	**A0A075AK05_9TREM**	–	**Uncharacterized protein**	***Opisthorchis viverrini***	**Calycin superfamily; fatty-acid binding protein (FABP) family**
**12**	TR21279|c0_g5	i1	161	C1LY00_SCHJA	–	Glutathione S-transferase (EC 2.5.1.18)	*Schistosoma japonicum* (blood fluke)	0
**13**	TR17688|c0_g1	i2	140	T1K7W8_TETUR	–	Uncharacterized protein	*Tetranychus urticae* (two-spotted spider mite)	0
**14**	TR21279|c0_g3	i8	192	Q06A71_FASHE	–	Glutathione transferase sigma class (EC 2.5.1.18)	*Fasciola hepatica* (liver fluke)	GST superfamily
**15**	TR21279|c0_g5	i1	181	C1LY00_SCHJA	–	Glutathione S-transferase (EC 2.5.1.18)	*Schistosoma japonicum* (blood fluke)	0
**16**	TR18466|c1_g2	i1	159	Q5D2M7_9TREM	–	Myoglobin 1	*Paragonimus westermani*	Globin family
**17**	**TR17138|c0_g1**	**i1**	**121**	**A0A074ZQZ2_9TREM**	–	**Uncharacterized protein**	***Opisthorchis viverrini***	**Calycin superfamily; fatty-acid binding protein (FABP) family**
**18**	TR17112|c0_g1	i1	123	Q25595_CLOSI	–	Putative glutathione transferase	*Clonorchis sinensis* (Chinese liver fluke)	GST superfamily
**19**	**TR18162|c0_g1**	**i1**	**327**	**A0A075AK05_9TREM**	–	**Uncharacterized protein**	***Opisthorchis viverrini***	**Calycin superfamily; fatty-acid binding protein (FABP) family**
**20**	**TR17982|c0_g1**	**i1**	**579**	**I2K1A3_DEKBR**	–	**Peptidyl-prolyl cis-trans isomerase (EC 5.2.1.8)**	***Brettanomyces bruxellensis*** **AWRI1499**	**Cyclophilin-type PPIase family**
**21**	**TR18162|c0_g1**	**i1**	**374**	**A0A075AK05_9TREM**	–	**Uncharacterized protein**	***Opisthorchis viverrini***	**Calycin superfamily; fatty-acid binding protein (FABP) family**
**22**	TR18466|c1_g2	i1	179	Q5D2M7_9TREM	–	Myoglobin 1	*Paragonimus westermani*	Globin family
**23**	TR16134|c0_g1	i2	174	G9P9T2_HYPAI	–	Uncharacterized protein	*Hypocrea atroviridis* (*Trichoderma atroviride*)	0
**24**	TR17219|c0_g1	i1	302	G4V7Z7_SCHMA	–	Malate dehydrogenase (EC 1.1.1.37)	*Schistosoma mansoni* (blood fluke)	LDH/MDH superfamily, MDH type 2 family
**25**	TR17170|c0_g1	i1	153	0	No annotations	na	na	na
**26**	TR17688|c0_g1	i2	176	T1K7W8_TETUR	–	Uncharacterized protein	*Tetranychus urticae* (two-spotted spider mite)	0
**27**	**TR17138|c0_g1**	**i1**	**69**	**A0A074ZQZ2_9TREM**	–	**Uncharacterized protein**	***Opisthorchis viverrini***	**Calycin superfamily; fatty-acid binding protein (FABP) family**
**28**	**TR17982|c0_g1**	**i1**	**426**	**I2K1A3_DEKBR**	–	**Peptidyl-prolyl cis-trans isomerase (EC 5.2.1.8)**	***Brettanomyces bruxellensis*** **AWRI1499**	**Cyclophilin-type PPIase family**
**29**	**TR18162|c0_g1**	**i1**	**317**	**A0A075AK05_9TREM**	–	**Uncharacterized protein**	***Opisthorchis viverrini***	**Calycin superfamily; fatty-acid binding protein (FABP) family**
**30**	TR17367|c0_g1	i1	924	G7YF68_CLOSI	–	Enolase	*Clonorchis sinensis* (Chinese liver fluke)	0
**31**	**TR17138|c0_g1**	**i1**	**69**	**A0A074ZQZ2_9TREM**	–	**Uncharacterized protein**	***Opisthorchis viverrini***	**Calycin superfamily; fatty-acid binding protein (FABP) family**
**32**	TR21565|c1_g1	i1	386	G4LWI3_SCHMA	–	Aldehyde dehydrogenase, putative (EC 1.2.1.5)	*Schistosoma mansoni* (blood fluke)	Aldehyde dehydrogenase family
**33**	TR15091|c0_g1	i1	167	Q4VVC2_CLOSI	–	Malate dehydrogenase (EC 1.1.1.37)	*Clonorchis sinensis* (Chinese liver fluke)	LDH/MDH superfamily; MDH type 1 family
**34**	TR18374|c0_g1	i1	92	S4UI50_FASHE	–	Triosephosphate isomerase (EC 5.3.1.1)	*Fasciola hepatica* (liver fluke)	Triosephosphate isomerase family
**35**	TR21279|c0_g3	i8	193	Q06A71_FASHE	–	Glutathione transferase sigma class (EC 2.5.1.18)	*Fasciola hepatica* (liver fluke)	GST superfamily
**36**	TR19268|c0_g1	i1	266	G7YAJ9_CLOSI	–	Tegument antigen	*Clonorchis sinensis* (Chinese liver fluke)	0
**37**	TR13906|c0_g1	i1	126	0	G4VAB2_SCHMA	Putative uncharacterized protein	*Schistosoma mansoni* (blood fluke)	0
**38**	TR15690|c0_g1	i2	68	C8CB63_FENCH	–	Fatty acids binding protein	*Fenneropenaeus chinensis* (fleshy prawn)	0
**39**	TR15690|c0_g1	i1	249	W5LYZ8_LEPOC	–	Uncharacterized protein	*Lepisosteus oculatus* (spotted gar)	0
**40**	TR19268|c0_g1	i1	186	G7YAJ9_CLOSI	–	Tegument antigen	*Clonorchis sinensis* (Chinese liver fluke)	0
**41**	TR19739|c0_g1	i1	312	G4V6X1_SCHMA	–	Sh3 domain grb2-like protein B1 (Endophilin B1)	*Schistosoma mansoni* (blood fluke)	0
**42**	TR21565|c1_g1	i1	211	G4LWI3_SCHMA	–	Aldehyde dehydrogenase, putative (EC 1.2.1.5)	*Schistosoma mansoni* (blood fluke)	Aldehyde dehydrogenase family
**43**	TR21722|c2_g1	i8	231	G7YFI1_CLOSI	–	3-oxoacyl-[acyl-carrier-protein] reductase	*Clonorchis sinensis* (Chinese liver fluke)	Short-chain dehydrogenases/reductases (SDR) family
**44**	TR17741|c0_g1	i1	725	B1NI98_FASHE	–	Heat-shock protein 70	*Fasciola hepatica* (liver fluke)	Heat-shock protein 70 family
**45**	TR19268|c0_g1	i1	95	G7YAJ9_CLOSI	–	Tegument antigen	*Clonorchis sinensis* (Chinese liver fluke)	0
**46**	**TR18162|c0_g1**	**i1**	**204**	**A0A075AK05_9TREM**	–	**Uncharacterized protein**	***Opisthorchis viverrini***	**Calycin superfamily; fatty-acid binding protein (FABP) family**
**47**	TR21565|c1_g1	i1	605	G4LWI3_SCHMA	–	Aldehyde dehydrogenase, putative (EC 1.2.1.5)	*Schistosoma mansoni* (Blood fluke)	Aldehyde dehydrogenase family
**48**	TR19308|c0_g1	i1	1131	G7YUU9_CLOSI	–	Chaperonin GroEL	*Clonorchis sinensis* (Chinese liver fluke)	Chaperonin (HSP60) family
**49**	TR19308|c0_g1	i1	513	G7YUU9_CLOSI	–	Chaperonin GroEL	*Clonorchis sinensis* (Chinese liver fluke)	Chaperonin (HSP60) family
**50**	No significant hits					na	na	0

*Abbreviation*: *na* not applicable

Those protein spots which had top matched hits to gene components in the in-house transcriptome assembly which were not annotated through Trinotate functional analysis or BLASTx searches of the matched contig sequence through the NCBInr database (2 spots in the somatic profile and 2 in the ES profile) may be previously unknown proteins which are present in paramphistomes or even unique to *C. daubneyi*. Matching of FPKM top gene components to the most abundant protein spot identifications seen with the generated proteomic data was seen with 5 separate gene component identifications (TR17138, TR18162, TR17982, TR22034 and TR20361). All 5 were identified in the ES profile, representing 17 of the top 50 protein spots, and 3 of these gene components (TR17138, TR18162 and TR17982) were identified in the somatic profile, representing 15 of the 50 most abundant proteins. Interestingly, TR17138 and TR18162, both identified as FABPs (CdFABP III and CdFABP IL2), constituted the vast majority of matches between gene components and putative protein identifications (13 of 17 identifications in the ES proteome and 13 of 15 identifications in the somatic proteome).

**Fig. 4:**
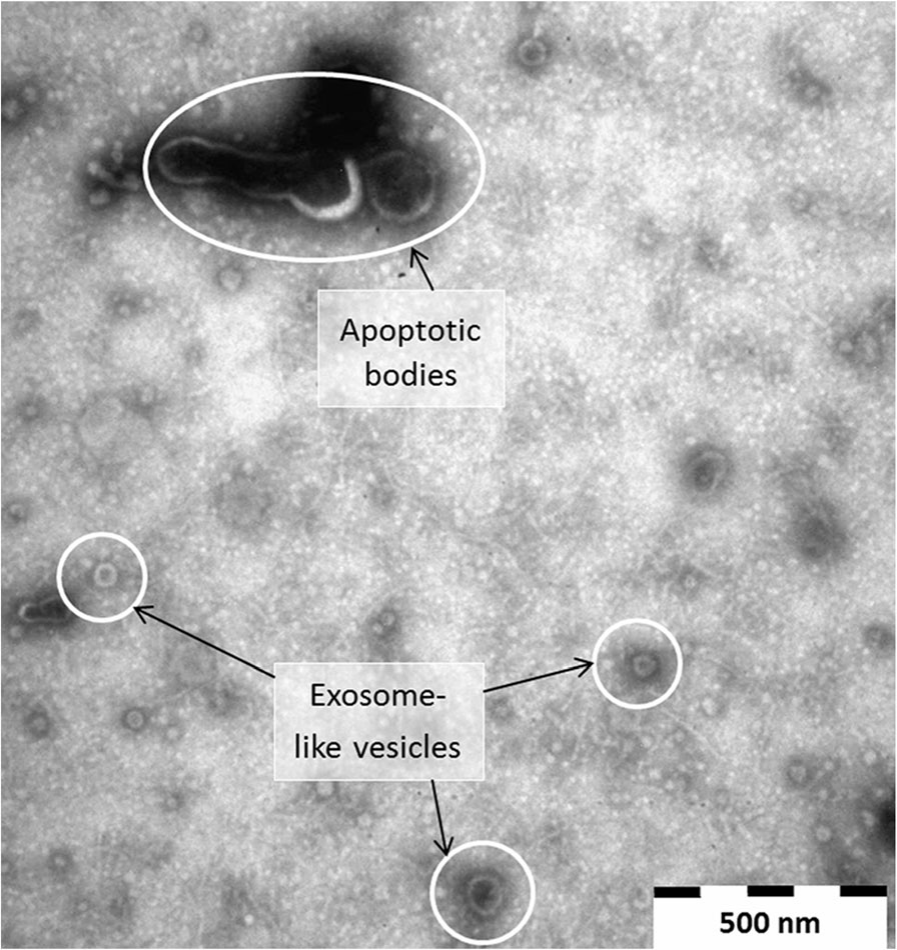
Extracellular (including exosome-like) vesicles (EVs) isolated from adult *C. daubneyi* excretory/secretory (ES). ES products were produced during *in vitro* culture and EVs identified using transmission electron microscopy. Preliminary vesicle characterisation (exosome-like and apoptotic bodies) are based on approximate size only

TEM imaging of vesicle enriched *in vitro* culture media samples indicated the release of extracellular vesicles (EVs) by rumen fluke parasites *in vitro* (Fig. 4). The likely presence of exosome-like EVs is indicated by the identification of appropriately sized vesicles ranging in diameter from 30 to 100 nm [39]. Of the top 50 most abundant proteins (Table 2) identified in the ES proteome profile 46% (23/50) contained proteins which have previously been identified as packaged and released in extracellular vesicles from helminth species or are found listed in the ExoCarta database [40] as highlighted in Table 2 and detailed further in Additional file 1: Table S4.

### Gas production study

Statistical analysis of calculated cumulative gas production volumes, ammonia concentration and protozoal counts did not identify any significant differences attributable to the presence of rumen fluke at either the 6 or 24-h time points. Significant differences were detected between the silage only (Group 4) and blank treatment (Group 1) bottles in respect of their protozoal counts at 24 h (Table 4), which was attributed to the respective presence/lack of grass silage fermentation substrate.

**Table 4:** *In vitro* gas production culture of adult *C. daubneyi.* Average concentrations of ammonia, VFAs and protozoan counts observed in batch culture vessels for each treatment group after 6 and 24 h *in vitro* culturing time points, along with 24 h average gas production volume data

Item	6-hour cultures							24-hour cultures						
	Silage	Silage + Fluke	Fluke	Blank	*F*	*P*	SE	Silage	Silage + Fluke	Fluke	Blank	*F*	*P*	SE
Total gas volume (ml)	–	–	–	–	–	–	–	7.46	7.05	4.17	4.01	1.62	0.237	1.444
Ammonia (mmol/l)	8.09	8.18	8.56	9.74	0.5	0.691	1.081	16.65	17.23	17.56	18.25	1.68	0.224	0.515
Total VFAs (mmol/l)	82.95^a^	85.79^a^	67.55^b^	66.05^b^	32.87	<0.001	1.784	103.95^a^	111.82^b^	81.92^c^	77.31^c^	109.3	<0.001	1.601
Acetate (mmol/l)	*53.11* ^*a*^	*54.75* ^*a*^	*44.43* ^*b*^	*43.68* ^*b*^	72.24	<0.001	0.676	*65.23* ^*a*^	*69.19* ^*a*^	*51.07* ^*b*^	*49.12* ^*b*^	87.01	<0.001	1.077
Proprionate (mmol/l)	*14.91* ^*a*^	*15.65* ^*a*^	*11.83* ^*b*^	*11.07* ^*b*^	17.59	<0.001	0.538	*18.44* ^*a*^	*21.21* ^*b*^	*14.90* ^*c*^	*12.76* ^*d*^	140.77	<0.001	0.316
Butyrate (mmol/l)	*10.77* ^*ab*^	*11.11* ^*b*^	*8.35* ^*c*^	*8.41* ^*ac*^	7.53	0.004	0.541	*13.68* ^*a*^	*14.66* ^*a*^	*10.80* ^*b*^	*10.52* ^*b*^	51.98	<0.001	0.287
Others (mmol/l)	*4.17* ^*a*^	*4.27* ^*a*^	*2.95* ^*b*^	*2.89* ^*b*^	11.81	<0.001	0.219	*6.60* ^*a*^	*6.75* ^*a*^	*5.14* ^*b*^	*4.91* ^*b*^	28.48	<0.001	0.1796
Protozoans (×10^5^/ml)	2.75	2.60	1.97	2.06	2.20	0.165	2.72 × 10^4^	2.54^*a*^	2.26^*ab*^	1.85^*ab*^	1.60^*b*^	4.98	0.018	1.86 × 10^4^

Superscripts denote the results of *post-hoc* testing using Bonferroni measures with multiple comparisons in a one-way ANOVA

*Abbreviation*: *SE* standard error of the mean

**Fig. 5:**
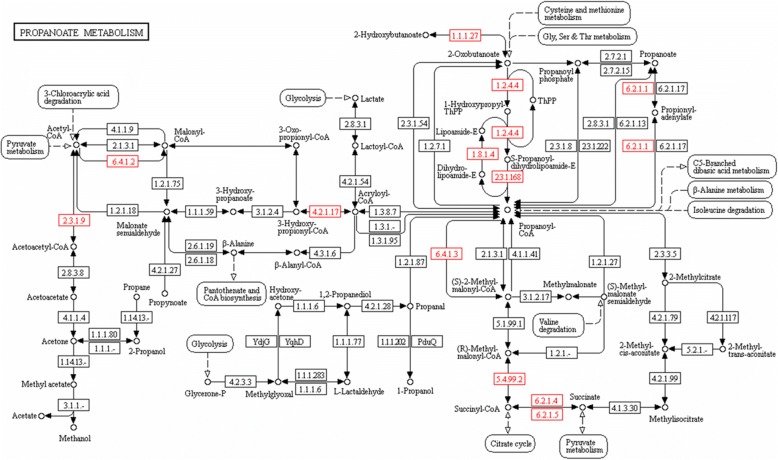
KEGG pathway map for propionate metabolism. EC codes identified in *C. daubneyi* transcript data based on those known in the helminth species *C. elegans* and *S. mansoni* available in the KEGG organism database are highlighted in red

With the measurements of VFA profiles, silage was seen to have a significant impact on VFA concentrations as expected, with significant differences seen between silage-positive and -negative groups at 6 h for total VFA concentrations (tVFAs), acetate, propionate and others. Butyrate was seen to be significantly different between group 3 (silage and rumen fluke) and both group 2 (fluke only) and group 1 (blank culture) bottles but not different to the silage only group (group 4). In addition, no significant difference was observed between the silage only (group 4) and blank culture groups (group 1) at 6 h. At 24 h, the same effect of silage treatment was evident, with significant differences between silage-positive and -negative groups for acetate, butyrate and other VFAs (Table 4). For the measurement of total VFAs a significantly higher concentration was seen in the silage and rumen fluke group (group 3) *vs* the silage only group (group 4) and both of these treatment groups compared to the silage negative bottles. This appears to be attributed to the significantly higher concentration of propionate detected in the fluke treated bottles *vs* their respective controls at 24 h. Analysis of the VFA profiles within each culture group revealed a significant increase in propionate in response to the presence of rumen fluke in both the silage positive and silage negative culture groups at 24 h. At 6 h, slightly higher propionate concentrations were recorded in the fluke treated groups in comparison to their respective silage/blank controls also but this was not statistically significant. Total VFA concentration was observed to be higher in the fluke only groups compared to the blank group, but again this was not significant in the measurement of total VFAs. Bioinformatic analysis supported rumen fluke propionate metabolism as a number of key genes present in the *C. daubneyi* transcriptome are involved in propionate production (Fig. 5).

## Discussion

The present study produced a transcriptome assembly that supported our first (and subsequent) global proteomic profiles of the ES and soluble somatic proteins of the rumen fluke *C. daubneyi*. In addition, this study shows for the first time that the presence of rumen fluke parasites impacts upon VFA production profiles in a rumen *in vitro* culture and may therefore impact on rumen fermentation kinetics in the host.

During transcriptomic analysis, signal peptides were predicted for 1.8% of mapped contigs, which is 2–3 fold lower than has previously been observed in other trematodes; with 4.1% of predicted proteins containing an signal peptide in an *Fascioloides magna* transcriptome [17] and 5.1% in an *Fasciola gigantica* transcriptome [46]. However, both Cantacessi et al. [17] and Young et al. [46] describe transcriptomes which, whilst also produced by *de novo* assembly, were sequenced on an Illumina Genome Analyzer II platform with the resulting assembly performed using alternative bioinformatics software tools; Oases and SOAPdenovo respectively. In addition, both transcriptomes also reported fewer numbers of unique contigs and mapped reads than currently described for *C. daubneyi*, highlighting that notable differences are often obtained with different sequencing and data analysis approaches which can make direct comparisons problematic [47]. Therefore, future genome-guided assemblies may allow for more accurate comparisons to be completed. Of note is the absence of an analysis regarding the contigs containing a signal peptide within the *P. cervi* transcriptome by Choudhary et al. [25].

Secreted proteins (as indicated by a signal peptide) are thought to play a crucial role in the biology of parasitic helminths [48] and particularly in host-parasite interactions [49]. Thus, although classical signal peptide based secreted proteins are of lower abundance in the *C. daubneyi* transcriptome, they were present in several highly expressed sequences, i.e. 12 of the 50 most expressed gene components contained a predicted signal peptide. In addition, rumen fluke may utilise non-classical communication host interaction pathways such as carrying proteins as cargo of extracellular vesicles [38] and to this end many previously associated EV proteins were identified in the *C. daubneyi* ES preparation.

Overall, the *C. daubneyi* ES proteome was similar to related fluke species including *F. magna* [17] and *F. hepatica* [32, 50], with FABPs, proteases and peptidase proteins, including cathepsins, present. Proteins belonging to the GST superfamily have been well studied in other helminths and are of interest for their role in drug detoxification and as potential vaccine candidates, as they are known to be immunogenic and immune modulators [51, 52], so the identification of these proteins in *C. daubneyi* is interesting for future studies. The GST profile identified in *C. daubneyi* appears to mirror that of fasciolids [28] with the identification of one zeta class and two omega class GSTs. Therefore, the presence of a zeta class GST in *C. daubneyi* no longer makes *Fasciola* unique as suggested previously [27]. Of significant interest is the apparent expansion of the sigma class GST protein family with multiple sequences identified in *C. daubneyi* compared to significantly fewer in the fasciolid liver flukes [28, 53]. It is likely that GSTs are present in the ES due to secretion via EVs as demonstrated for *F. hepatica* [37] and therefore this expansion of Sigma-like GSTs may be more related to host and regulation and regulation of the rumen environment rather than xenobiotic detoxification.

Additional detoxification proteins, namely CYP450s, were also identified in the *C. daubneyi* transcriptome but, as expected, were not represented in the abundant proteome. The expression of a single monooxygenase and one reductase mirrors *F. hepatica* [27] and the absence from a proteomic studies, including those incorporating membrane proteomics, has also been noted in other helminths [54]. However, it still remains likely that CYP450s play an important role in fluke biology and xenobiotic detoxification.

Many of the 50 most abundant proteins identified by LC MS/MS in both the ES and somatic profiles are described as uncharacterised, although the majority of hits achieved were to proteins found in other fluke species or invertebrate animals. In contrast to the published ES 2DE proteome of *F. hepatica* [50], the profile observed for adult *C. daubneyi* parasites is not dominated by a few major proteins, but has numerous proteins present across a wide pH and MW range. A diverse profile of ES proteins is likely related to feeding, interaction and communication with the diverse niche of the rumen inhabited by adult rumen fluke.

With the complex nature of the rumen environment the decision to capture transcripts immediately ex-host opens the dataset to contamination from associated rumen eukaryotic microbes or plant tissue which was not removed during the wash process described. It is also noted that protozoan organisms have previously been observed within the oesophagus of rumen fluke parasites examined under SEM [55]. However, only 2 contigs in our dataset were found to have a best match to bovine sequences indicating a likely host contamination, but with only 2 such hits from over 73K contigs this suggests host contamination is minimal. Bacterial sequences should have been largely excluded from our sequencing effort by the poly-A enrichment step during sequencing library preparation as mRNA from eukaryotic organisms is poly-adenylated to add stability whereas polyadenylation in prokaryotes is minimal in length and rapidly degraded [56]. However, it cannot be excluded that some erroneous protozoan, fungal and plant sequences are present in the dataset. That being said, a significant proportion of the most highly expressed sequences align to other trematode parasites.

This new rumen fluke data may have a role in developing future diagnostics as it identified both genes potentially unique to *C. daubneyi*, or to paramphistome species, and novel proteins in the ES profile which are likely to be host-exposed and potentially antigenic. This potential biomarker panel could be tested for the potential to detect paramphistome-specific DNA or protein signatures in faecal samples or antigen/antibody detection in either faecal or blood based ELISA tests such as those which have been developed for *F. hepatica* infections [57, 58].

During the life-cycle of *C. daubneyi* in the definitive host, described by Devos et al. [59], it is not yet clear how long a mature infection potentially persists. Moreover, different hypotheses have been formulated for how rumen fluke parasites feed in the host. Choudhary et al. [25] suggested that rumen flukes may survive by absorbing blood glucose from the host at the site of attachment to the rumen wall, but given the superficial nature of the attachment of their acetabulum, with the oral sucker exposed to the rumen contents this is thought unlikely. However, the present gas production experiment successfully culturing rumen fluke for 24 h ex-host in a rumen fluid-buffer mix provides evidence that rumen fluke are likely to prey on the rumen microbial community and/or obtain their nutrition from the products of microbial fermentation occurring in the rumen or the abundant plant material present as digesta, although this requires further investigation. The high abundance of uncharacterised FABPs in each proteome profile (both ES and somatic), with high levels of transcription also seen, suggests that fatty acids are of significant importance in paramphistome biology. The rumen appears a highly appropriate niche for these parasites to select, as it is known that many trematodes cannot synthesise their own fatty acid complement [60, 61]. Such an environment is likely to have driven the expansion of the FABPs in *C. daubneyi*. This is especially so given that this expansion is driven in the designated CdFABP IL groups 1 and 2. Both of these FABP groups clustered with the vertebrate ileal and liver FABPs. Vertebrate ileal and liver FABPs are noted for their ability to bind fatty acids and bulky ligands such as cholesterol. Therefore, with the levels of fatty acids and cholesterol esters found within the rumen [62] that these groups of *C. daubneyi* FABPs have expanded to exploit this niche.

Understanding the impact of rumen fluke on the host is crucial. Thus, the present study utilised an *in vitro* experiment to understand rumen dynamics. Interestingly, no differences in protozoan numbers were observed despite the suggestion of predation on protozoans by adult rumen flukes. The only significant difference detected in this gas production experiment was an increase in propionate and total VFA production. It has been established that several nematode, cestode and trematode species actively produce propionate as an end product of their metabolism *via* the malate dismutation pathway, including succinate decarboxylation under anaerobic conditions, and that the presence or absence of oxygen appears to have no effect on their survival and ability to metabolise energy [63, 64]. In the malate dismutation pathway, redox balance is maintained when twice as much succinate as acetate is formed, with succinate then being further converted to propionate by a decarboxylation reaction [65], with acetate and propionate the main excretory products produced by adult *F. hepatica* metabolism.

Given the increase in rumen fluke cultures of propionate levels it is likely that there may be a shift in the resulting microbial population. Thus, it is beneficial to confirm the source of propionate. Bioinformatics analysis here supports the hypothesis that the higher propionate levels detected over a 24-h *in vitro* rumen fermentation experiment in vessels with the addition of rumen fluke parasites is likely due to the production of propionate *via* rumen fluke metabolic activity. A number of key genes involved in the succinate decarboxylation pathway, and evidence of a complete pathway to propionate *via* genes involved in valine, leucine and isoleucine degradation (EC 1.2.4.4, 1.8.1.4 and 23.1.168 in Fig. 5) were identified in the *C. daubneyi* transcript data, with evidence of active gene expression from samples captured directly from the natural rumen environment.

However, although the greater levels of VFAs measured, and specifically propionate, will contribute to host nutrition, the ratio and concentration of rumen VFAs is also an important factor in the development of rumen acidosis. Where propionate levels increase and the acetate: propionate ratio decreases it is known that acidotic conditions in the rumen may occur. This is generally linked to levels of fibre *vs* starch based feeds in the diet [66], but in animals where the diet fed creates higher levels of propionate in the rumen with pH conditions bordering acidosis, any additional production of propionate linked to the presence of rumen fluke could be an important factor to consider where acidosis then occurs. Lower acetate: propionate ratios are also linked to decreases in methane emissions from the rumen [67], which is important given methane emissions are a significant source of energy loss in ruminant systems, and also an important greenhouse gas [68].

Results of an abattoir study [69] identified significantly lower carcass cold weight and fat coverage measurements for rumen fluke infected beef cattle in comparison to their helminth-free counterparts, indicating a potential interaction between rumen fluke infections in temperate climates with *C. daubneyi* and measures of animal production. Additionally, with evidence of inflammatory reactions and atrophy of the rumen papillae previously identified in association with *C. daubneyi* infection [70], the effects of chronic tissue inflammation on the host animal associated with heavy and prolonged rumen fluke burdens, and atrophy of the rumen papillae and any potential for reducing the surface area available for nutrient uptake is unknown.

## Conclusions

The present study provided a discovery platform (transcriptome, proteomes, EV isolation pipeline and *in vitro* fermentation system) to study the *C. daubneyi* host-interaction. This work has highlighted the FABPs as key players in survival within the rumen environment. Furthermore, the impact of adult fluke on rumen functionality has been demonstrated with reduced acetate: propionate ratio suggesting that acidotic conditions may occur within the rumen. However, further investigation into how the presence of rumen fluke infections may impact on animal health and production measures in temperate climates is clearly needed.

## Additional files

Additional file 1: Full polyomic data. **Table S1.** Top *C. daubneyi* transcriptomic hits. **Table S2.** The full *C. daubneyi* transcriptomic hits. **Table S3.** BLAST searches of *C. daubneyi* transcripts against *P. cervi*. BLAST searches of *C. daubneyi* transcripts. **Table S4.** The full analysis of putative protein identifications from the excretory/secretory proteome profile of adult *C. daubneyi*. **Table S5.** The full analysis of putative proteins identified from the soluble somatic proteome profile of adult *C. daubneyi*. (XLSX 12697 kb)

Additional file 2: **Figure S1.** Phylogenetic analysis of *C. daubneyi* fatty acid binding proteins. Neighbor-joining phylogenetic tree constructed using amino acid sequences through MEGA v 6.0 with 1000 bootstrapped support and a Poisson correction. All reported accession numbers are from Genbank. Where sequences were identified *in silico*, only gene product numbers are reported. (TIF 1007 kb)

## Abbreviations

BLAST: Basic Local Alignment Search Tool
DMEM: Dulbecco’s modified eagle medium
ES: Excretory/secretory
FABP: Fatty acid binding protein
FEC: Faecal egg count
FPKM: Fragments per kilobase of transcript per million mapped reads
GO: Gene ontology
GST: Glutathione transferase
MS/MS: Tandem mass spectrometry
MWCO: Molecular weight cut-off
PTMs: Post-translational modifications
TEM: Transmission electron microscopy
VFA: Volatile fatty acids
