# Supplementary figures and images for: Polyomic tools for an emerging livestock parasite, the rumen fluke Calicophoron daubneyi; identifying shifts in rumen functionality
Source: Parasit Vectors. 2018 Dec 4;11:617. doi: 10.1186/s13071-018-3225-6 (PMC6278170; doi:10.1186/s13071-018-3225-6)

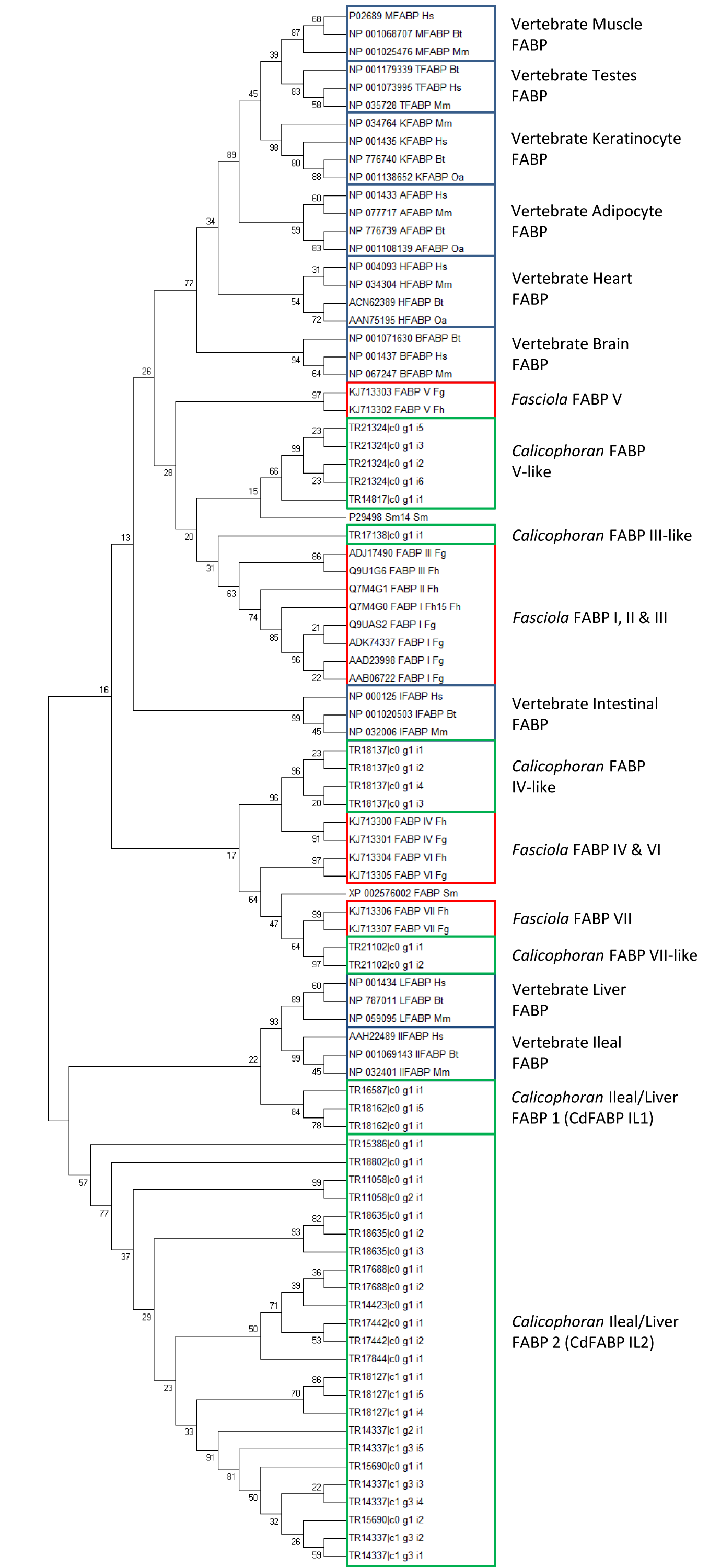

Supplement: Supplementary file 2 — Figure S1. Phylogenetic analysis of C. daubneyi fatty acid binding proteins. Neighbor-joining phylogenetic tree constructed using amino acid sequences through MEGA v 6.0 with 1000 bootstrapped support and a Poisson correction. All reported accession numbers are from Genbank. Where sequences were identified in silico, only gene product numbers are reported. (TIF 1007 kb) [file 13071_2018_3225_MOESM2_ESM.tif]
